# Impact of Solid Materials in the Gap Space between Driving Electrodes in a MEMS Tri-Electrode Electrostatic Actuator

**DOI:** 10.3390/s24092743

**Published:** 2024-04-25

**Authors:** Mehdi Allameh, Byoungyoul Park, Cyrus Shafai

**Affiliations:** 1 Department of Electrical and Computer Engineering, University of Manitoba, Winnipeg, MB R3T 5V6, Canada; cyrus.shafai@umanitoba.ca; 2 Quantum and Nanotechnologies Research Centre, National Research Council Canada, Edmonton, AB T6G 2M9, Canada; byoungyoul.park@nrc-cnrc.gc.ca

**Keywords:** MEMS, electrostatic actuator, pull-in effect, microwave applications, voltage reduction, microfabrication, charge analysis, tri-electrode actuator

## Abstract

MEMS electrostatic actuators can suffer from a high control voltage and a limited displacement range, which are made more prevalent by the pull-in effect. This study explores a tri-electrode topology to enable a reduction in the control voltage and explores the effect of various solid materials forming the space between the two underlying stationary electrodes. Employing solid dielectric material simplifies fabrication and can reduce the bottom primary electrode’s fixed voltage. Through numerical analysis, different materials were examined to assess their impact. The results indicate that the primary electrode’s fixed voltage can be reduced with an increase in the dielectric constant, however, with the consequence of reduced benefit to control voltage reduction. Additionally, charge analysis was conducted to compare the actuator’s performance using air as the gap-spacing material versus solid materials, from the perspective of energy conservation. It was found that solid materials result in a higher accumulated charge, reducing the need for a high fixed voltage.

## 1. Introduction

Micrometer-sized actuators, especially those based on microelectromechanical systems (MEMSs), have found extensive applications in various fields, such as biomedical devices, chemical sensors, CMOS-MEMS sensors, optical sensors, and switches, due to their miniature size and capabilities [1,2,3,4,5,6,7]. One of the preferred types of MEMS actuators [8,9,10,11,12,13,14] is the electrostatic actuator, known for its simplicity in terms of fabrication and its ability to generate significant force without requiring a high steady-state current. This makes electrostatic actuators particularly suitable for environments where heat is a concern, like optical sensors or silicon photonics devices [15,16,17,18,19].

However, electrostatic actuators face challenges due to their requirement for relatively high control voltages, which can be a limiting factor in certain applications. To address this, researchers have proposed various approaches. In [20], a design and testing methodology for a laterally movable electrostatic actuator, specifically aimed at RF MEMS switches, was proposed. This actuator was developed to operate efficiently at a low voltage level. Similarly, in [21], a low voltage soft actuator was introduced, which was tailored for applications in human interface machines. In another approach, a silicon photonics MEMS switch was designed and fabricated, capable of operating at as low as 3.75 V [22]. Shunti et al., in [23], focused on the introduction of an RF MEMS shunt switch that featured a low pull-in voltage of 5.2 V, achieved through the use of U-shaped meanders. Zhu and Pal, in [24], explored the application of combined electrostatic and electrothermal actuation to create a highly reliable RF MEMS switch that operates at low voltages. However, this approach necessitated a relatively high temperature for the electrothermal actuation. In [25], a different method of internal dielectric transduction was used to reduce the pull-in voltage from 1.27 V to 0.62 V. All of the solutions mentioned are very unique, tailored to the specific application and fabrication method. Furthermore, as mentioned above, they only address the high voltage problem and do not necessarily consider the limited travel range.

However, in regard to the tri-electrode actuator topology [26,27,28], the aim is to reduce the control voltage and also improve the range of motion. This method can be applied to existing parallel-plate electrostatic actuator design, with very minor modifications in the design and fabrication. Also, to further optimize this setup, a more advanced design [29,30] incorporates a solid material that bridges the gap between the stationary electrodes, effectively reducing the fixed voltage needed to operate the actuator. The addition of a solid material to support the region between the two underlying stationary electrodes simplifies fabrication, as only the MEMS actuator itself now needs to be released from the substrate.

This article delves into the exploration of different materials and their potential impact on the design of tri-electrode actuators. By examining the effects of various dielectric materials on the actuator’s functionality, the aim was to provide valuable insights that could lead to significant improvements in the design and performance of this actuator. In Section 2 of our study, an introduction to the fundamental principles behind tri-electrode actuators is provided, including their operation and the mechanisms involved. A comprehensive numerical analysis is detailed in Section 3, where the impact of various materials on the performance of tri-electrode actuators is explored. By creating simulations with different materials and setups, the aim was to understand the intricacies of how these changes affect the actuator’s output. Building upon the analytical framework established in Section 3, the Section 4 presents a discussion of the results obtained from our simulations. Finally, in the concluding Section 5, our key findings are summarized.

## 2. Tri-Electrode Topology

The pull-in effect refers to the constraint imposed on the motion of electrostatic actuators due to their typical parallel-plate structure, which restricts displacement to about one-third of the original gap between the actuator’s electrodes. While numerous solutions exist to mitigate this effect, they often fail to adequately reduce the high control voltage inherent in these systems. Among these methods, the tri-electrode configuration [26] stands out due to its potential to significantly decrease the control voltage, while also resolving the pull-in effect.

### 2.1. Tri-Electrode Configuration

In tri-electrode configurations, a MEMS moving electrode is present, which can be grounded. Additionally, there are two fixed electrodes, each receiving a voltage from independent sources. One of these fixed electrodes (intermediate electrode) has a perforated structure and is situated near the MEMS (moving) electrode, creating a gap that provides operational space for the MEMS electrode. The perforated electrode is subjected to a variable voltage, which directly influences the actuator’s movement. The second fixed electrode (primary electrode) is located farther from the MEMS electrode and establishes a background electric field using a higher voltage.

**Figure 1:**
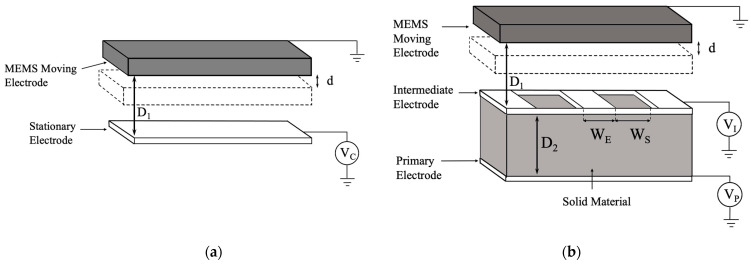
A 3D schematic of conventional and tri-electrode electrostatic actuator configurations. (**a**) Conventional parallel-plate actuator topology with one MEMS moving electrode and one stationary electrode. (**b**) Tri-electrode topology with one MEMS moving electrode and two stationary electrodes, one perforated (intermediate electrode) and one solid (primary electrode).

As illustrated in Figure 1, the conventional electrostatic actuator schematic is contrasted with the tri-electrode design, emphasizing the differences in their geometric structure. In order to study the actuator’s performance, the parameters are defined in Table 1.

The electrostatic force between the conventional parallel-plate actuator is well-documented in the literature. However, since the fringing fields between the MEMS electrode and fixed electrodes play a key role in the performance of the tri-electrode topology, a representation of Gauss’s law was employed to investigate the tri-electrode’s performance [26].

The performance of a tri-electrode actuator was investigated through the application of finite element analysis (FEA) and the restoring spring force method (RSFM). COMSOL Multiphysics 6.2 software was employed for the FEA analysis. This methodology involved the quantification of the electrical force experienced by the MEMS electrode, as it was subjected to a range of voltages applied to the intermediate electrode, while maintaining a constant voltage at the primary electrode. The analysis encompassed the full range of displacements that the MEMS electrode could achieve in its movement towards the intermediate electrode. According to the RSFM method, the MEMS moving electrode should be designed with a linear spring constant. The MEMS can be designed either to have only a linear spring constant, or to limit the actuator performance within the displacement range, where the spring constant is linear. In this linear actuator, the response curves were derived to represent the displacement of the MEMS electrode in response to the control voltage applied to the intermediate electrode. These curves, explained in Section 2.2, provide insights into the understanding of the actuator’s performance characteristics.

### 2.2. Tri-Electrode Response Curve

**Figure 2:**
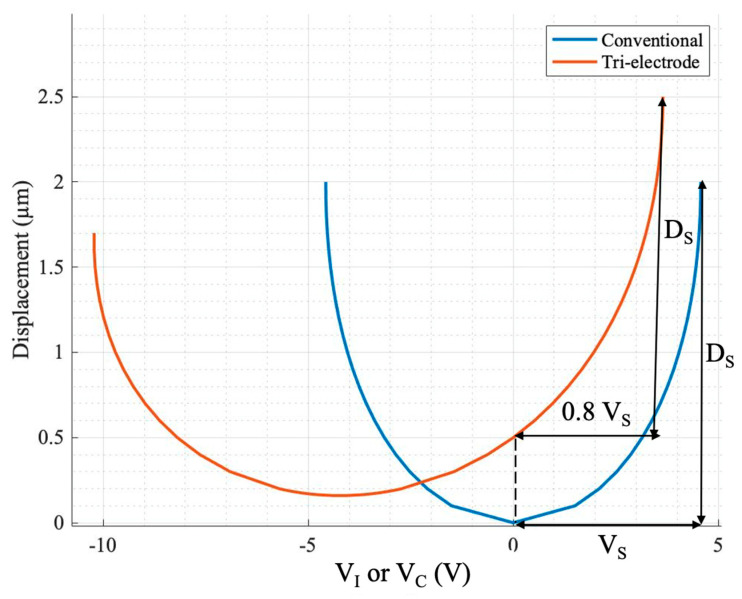
Displacement vs. control voltage (V_I_ or V_C_) for both conventional and tri-electrode configurations. The tri-electrode response curve is characterized by a unipolar power supply and a positive voltage range.

In order to investigate the actuator’s performance, response curves (displacement versus control voltage) were derived for different design parameters. All the results were compared to the conventional topology to observe the amount of improvement in the actuator’s operation. Thus, all the tri-electrode parameters were normalized to the conventional parameters. As explained, the displacement of a conventional actuator is limited to one-third of the initial gap due to the pull-in effect. The control voltage at which this snap down occurs is called the snap-down (pull-in) voltage (V_S_), and the corresponding displacement is called the D_S_. Figure 2 illustrates the response curve for both the conventional and tri-electrode configurations for an arbitrary tri-electrode configuration. The differences are apparent in terms of the smaller control voltage required for the same amount of displacement. The tri-electrode configuration specifications are summarized in Table 2. In this example, the conventional topology, with a D_1_ gap spacing of 6 µm, exhibits characteristic parameters of D_S_ = 2 µm and V_S_ = 4.58 V. The tri-electrode topology has the same D_1_ gap spacing as the conventional topology. The figure of merit (FOM) is a parameter defined to monitor the reduction in the control voltage, which is the displacement per unit voltage applied to the actuator, as shown below:

FOM = Displacement/Control Voltage range(1)

For the conventional topology, this is demonstrated using FOM_S_ (D_S_/V_S_). In the tri-electrode configuration, FOM is normalized to FOM_S_ to illustrate the performance improvement of the tri-electrode compared to the conventional topology. All the geometrical parameters are normalized to the gap spacing between the MEMS and the intermediate electrode (D_1_). This normalization allows the solutions derived to be applicable to tri-electrode actuators of various sizes. The response curve parameters, including the snap-down voltage and displacement, are depicted on the graph for both the conventional and tri-electrode setups.

As shown in Figure 2, the control voltage is smaller (0.8 V_S_) for the tri-electrode compared to the conventional topology (V_S_) for the same displacement (D_S_), demonstrating a higher FOM than the FOM_S_ of the conventional topology. Although this is not the best or most optimized design, it is presented here to illustrate the reduction in the control voltage using the tri-electrode topology. The positive voltage range was only examined in this graph, as it represents a higher level of performance than the conventional setup. This is referred to as the unipolar mode. If the control voltage for the same range of positive voltage were extended into the negative range, it would be called the bipolar mode. In order to analyze the tri-electrode actuator, three modes are defined. Two of these modes are the aforementioned unipolar and bipolar modes, which are used to study the control voltage reduction in terms of the applied power supply. Another mode is called maximum displacement, which addresses the issue of the pull-in effect by focusing on enhancing the displacement range before snap down. These modes are further detailed in [26,27].

### 2.3. Tri-Electrode Charge Analysis

**Figure 3:**
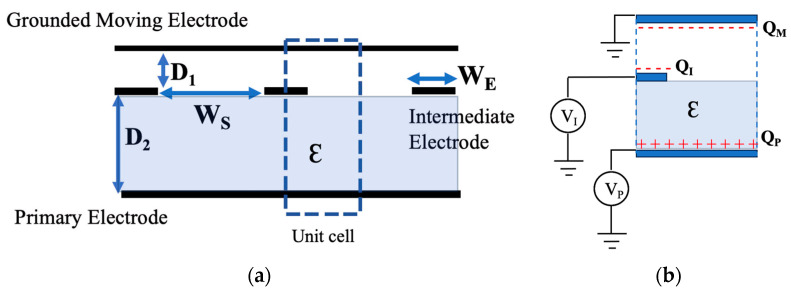
A 2D cross-cross section schematic of the tri-electrode actuator. (**a**) Highlight of the unit cell as the smallest repeatable part of the schematic. (**b**) The labeling of the unit cell. Q_P_, Q_I_ and Q_M_ show the surface charge density of the primary, intermediate and MEMS electrodes, respectively. This is for the case of V_P_ > V_I_ > 0.

In order to understand the functioning of the tri-electrode, the surface charge density of all three electrodes is investigated. This will provide insights into how the actuator performs. The studied configuration is chosen based on the design in Table 2, which is further discussed in the following sections. Figure 3 illustrates a 2D cross-section of the tri-electrode configuration. To expedite the analysis, a unit cell is defined as the smallest repeating section of the geometry. A unit cell is highlighted in Figure 3b, showing each electrode’s electric charge with different labels.

**Figure 4:**
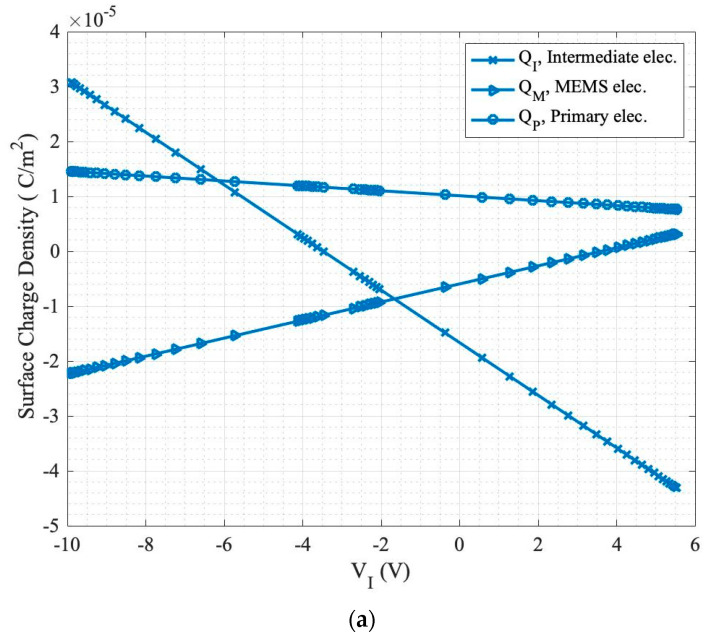

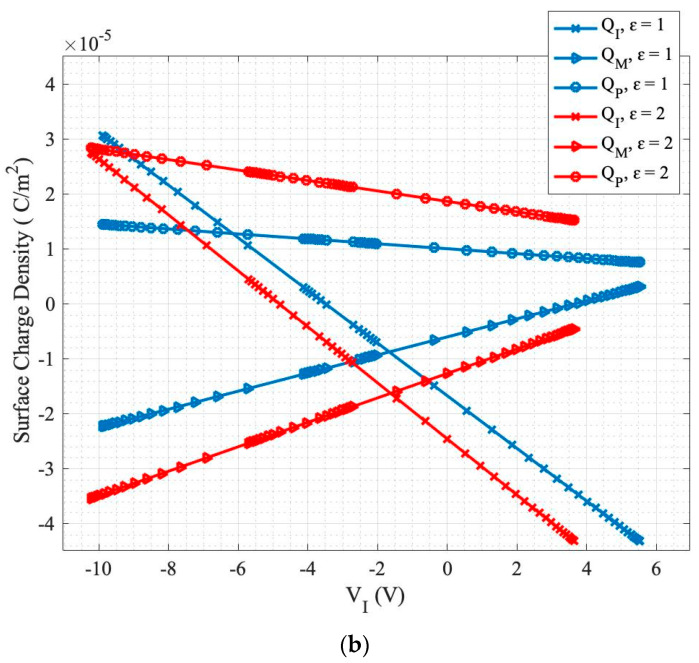
Surface charge density of the tri-electrode electrodes for: (**a**) air gap-spacing material when ɛ = 1 and (**b**) when ε = 1 and 2.

The surface charge density of each electrode is plotted in Figure 4, with the same design parameters as summarized in Table 2. The FEA analysis was utilized to determine the charges of each electrode. This study can help us understand the importance of a material with a dielectric constant larger than one, from a charge and stored energy perspective. According to Gauss’s law, the total charges of all three electrodes are always equal to zero. Therefore, studying the surface charge density on each electrode will explain the actuator’s response curve (displacement vs. V_I_ control voltage), according to the law of conservation of energy.

In order to plot Figure 4, the intermediate electrode voltage is swept, while the voltage applied to the primary electrode is considered fixed. At each V_I_ voltage, the total accumulated charge on each of the electrodes is calculated and divided by the electrode’s area. Then, the calculated surface charge density is plotted for all the single voltages applied. Regarding the boundary conditions of the study, a zero-charge condition was applied to the exterior boundaries in the simulations in order to prevent any displacement field from penetrating the boundary condition of the defined unit-cell geometry [26].

In Figure 4a, air (ε = 1) is considered as the material in the region between the intermediate and primary electrodes. Comparing the trend of the surface charge density on each electrode, it can be seen that by increasing the intermediate electrode voltage V_I_ from −10 to 5.5 V, the following occurs:

- The charge on the primary electrode declines, but always stays positive.
- When comparing the stored surface charges on the MEMS and primary electrodes (shown in Figure 4a), it can be seen that the charges have opposite polarities. The highest difference occurs at V_I_ = −10 V, which is the negative snap-down voltage. As V_I_ increases toward 5.5 V, the positive snap-down voltage, the charge values converge to a very close value. This observation suggests that increasing V_I_ results in a greater proportion of electric forces being directed towards the MEMS electrode, while less energy is dissipated within the solid material separating the intermediate and primary electrodes.
- The intermediate electrode’s surface charge density declines at a higher rate than the other two electrodes’ changes. As it is a smaller electrode, it should accumulate charges faster to balance the overall zero charge.
- For comparison, the surface charge density of a conventional two-electrode topology exhibits identical values for the charge per unit area at ±V_I_, yet with opposite polarities. This can be concluded from the analytical solutions in the literature.

Looking closely at the graph in Figure 4a, it can be concluded that when the applied control voltage (V_I_) is positive, the electric field generated by the primary electrode reaches the MEMS electrode more effectively than when V_I_ is negative. This occurs because the primary electrode is also positive, and both voltages (V_I_ and V_P_) share the same polarity. This characteristic represents the fundamental advantage of the tri-electrode topology over conventional systems. Additionally, as depicted in the tri-electrode’s orange colored response curve in Figure 2, the positive snap-down voltage (the maximum possible positive voltage before snap down) is lower than the conventional snap-down voltage, while the negative snap-down voltage is higher [26].

Figure 4b illustrates the surface charge density of the electrodes when there is a solid gap-spacing material with ε = 2 in the region between the intermediate and primary electrodes. Comparing the results with air, the trends are the same, but the charges accumulated on the electrodes when the same V_I_ > 0 are higher, resulting in a higher electrostatic force applied to the MEMS. Thus, when the same force is applied to the MEMS electrode, it is possible to apply a smaller primary voltage. Employing a material with a higher dielectric constant as the gap-spacing material can provide more energy toward the MEMS electrode from the fixed electrodes. However, the higher force can adversely affect the pull-in effect, resulting in a snap down at a larger control voltage (smaller FOM). The response curves are extracted for a series of different materials in the following section.

## 3. Numerical Analysis

The numerical analysis for the tri-electrode actuator using different gap-spacing materials is presented in this section. RSFM and FEA were employed to extract the response curves. 

In the RSFM method, the electrostatic force applied to the MEMS electrode is calculated using COMSOL across a wide range of control voltages (V_I_) and distances travelled between the intermediate and MEMS electrode (d). In the FEA calculations, the MEMS electrode is considered fixed at a distance d from the intermediate electrode. Then, the electrostatic forces are calculated as V_I_ is varied, while maintaining a fixed V_P_ voltage. This step is repeated for a different range of d, allowing for the acquisition of the electrostatic force on the MEMS electrode for all combinations of d and V_I_. In the next step, the restoring spring force is calculated, assuming a linear spring with a linear spring constant (F = −kd), where k is the spring constant, and d is the desired distance traveled. The MEMS moving electrode stops moving when the electrostatic and restoring spring forces balance at each applied V_I_, establishing a mechanically stable position. Therefore, the displacement corresponding to the applied V_I_ can be found. This is essentially how the RSFM method employs the FEA COMSOL 6.2 software to extract the response curve. MATLAB R2021a software was also used to implement this method. The response curve of the actuator can then be extracted at the desired V_I_ voltages [26].

Subsequently, FOM, the corresponding V_P_, and the maximum possible displacements were extracted from the response curves. The materials used in our study are reported in Table 3. The materials were chosen based on their common use in applications, specifically in RF, microwave, and printed circuit boards. Fortunately, a vast array of materials with other dielectric constants falls within the range of 1–10.2, which are suitable for a wide range of applications. Therefore, this study can be applied to any materials with a dielectric constant within this range.

**Figure 5:**
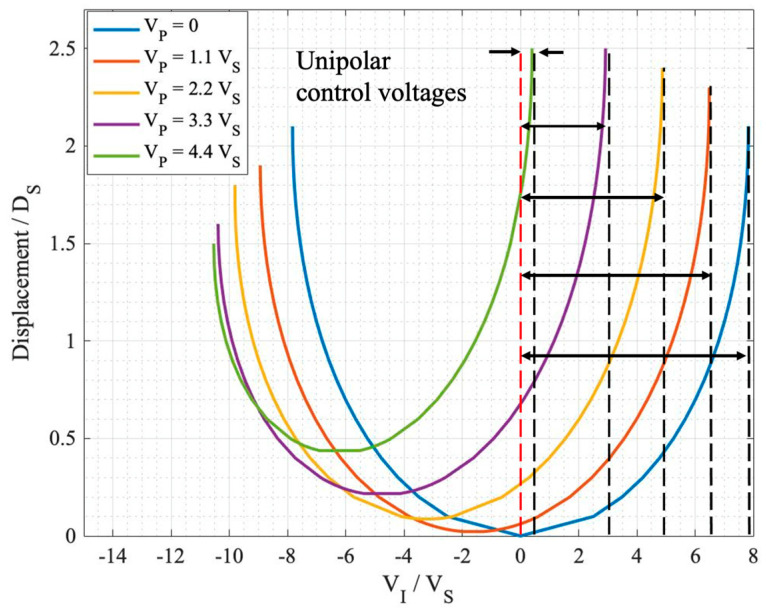
Displacement vs. control voltage (V_I_) for when ε = 2 and W_S_ = 3 W_E_ (Table 2) for five V_P_ values. Control voltages are highlighted for the unipolar mode.

The response curves are extracted by first sweeping the primary voltage (V_P_) from zero to a sufficiently high value. This value is deemed acceptable by the designer, as it can accommodate the specific design without limiting the actuator’s performance. The intermediate voltage (V_I_) is swept from negative to positive voltage ranges at each selected V_P_. The displacement of the MEMS electrode at each V_I_ control voltage is extracted and plotted as a point on the response curve (Figure 5). 

**Figure 6:**
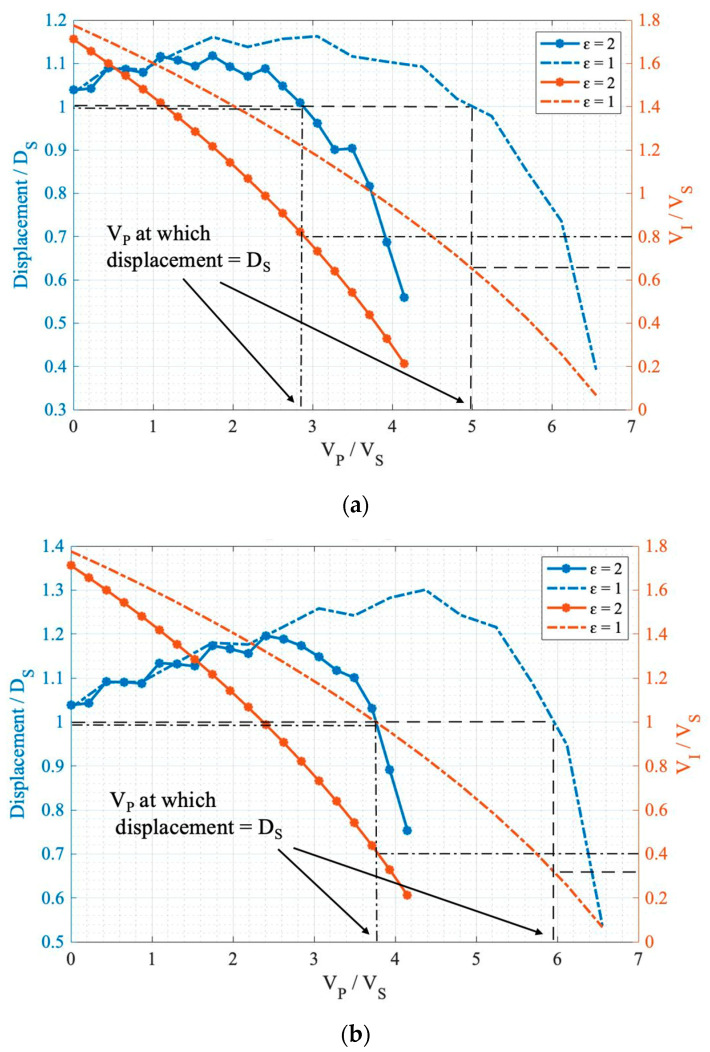
Displacement (left y-axis) and control voltage V_I_ (right y-axis) vs. V_P_ are plotted for: (**a**) unipolar and (**b**) bipolar modes. Each of the dashed lines is for ε = 1 or 2.

The aim is to find a V_P_ at which the displacement equals the conventional displacement of D_S_ (Figure 6), which would make the comparison between the tri-electrode and conventional actuator meaningful. This would allow the progress made in regard to the tri-electrode’s performance to be recognized by normalizing it in regard to the conventional actuator. Figure 5 shows the response curves for Teflon at various V_P_ values, consisting of five curves that clearly demonstrate the change in the response curve as V_P_ increases. 

For each curve in Figure 5, there is a specific displacement before snap down, either in the unipolar or bipolar modes. According to the definition of the modes [26], each mode’s displacement is plotted in Figure 6 for air (ε = 1) and Teflon (ε = 2) to understand the change in the control voltage and the displacement in one graph. The x-axis shows the sweeping V_P_, and the y-axis (left) is the displacement in each mode, corresponding to each of the curves in Figure 5. The y-axis (right) is the V_I_ control voltage. 

The displacement curve (left y-axis) is used to find the V_P_ at which displacement equals D_S_ and the V_I_ curve (right y-axis) is used to calculate the FOM normalized to FOM_S_, which is (V_I_/V_S_)^−1^. It can be concluded, as follows:

FOM/FOM_s_ = (D_S_/V_I_)/(D_S_/V_S_) = (V_I_/V_S_)^−1^ (2)

**Figure 7:**
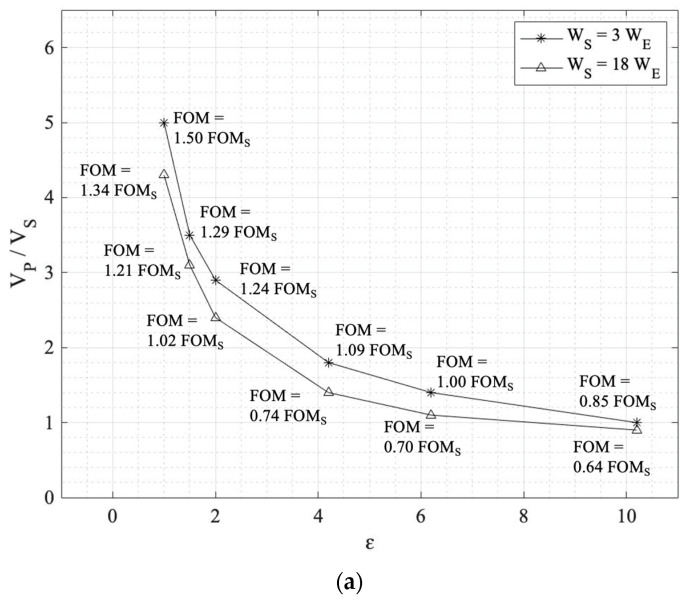

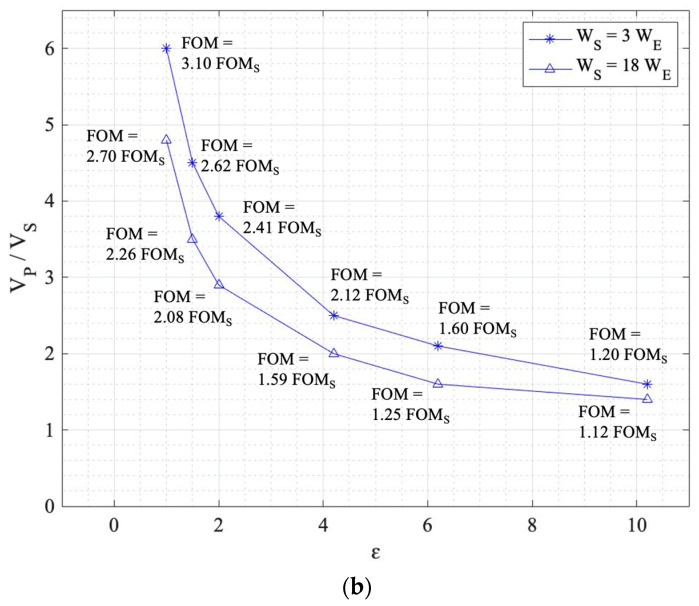
Primary electrode voltage (V_P_) vs. dielectric constant (ε) for: (**a**) unipolar and (**b**) bipolar modes.

The results of the numerical simulations are reported in Table 4 for two different intermediate electrode perforation ratios of (W_S_ = 3 W_E_ and W_S_ = 18 W_E_). With this, we are able to study the impact of different intermediate electrode spacing (W_S_) sizes with different solid gap-spacing materials in the D_2_ gap region. Figure 7 illustrates the primary electrode voltage (V_P_) versus the dielectric constant for all three modes to understand the impact of solid materials on reducing the primary electrode voltage more clearly.

## 4. Discussion

Employing solid materials as gap-spacing materials simplifies the fabrication of the tri-electrode configuration, since the intermediate electrode does not need to be suspended over the primary electrode. Providing a hollow area beneath the intermediate electrode complicates the manufacturing of the actuator from a microfabrication point of view. Additionally, using different materials, such as those available and commonly used in microwave and RF applications, demonstrates how they can be beneficial in the tri-electrode topology to reduce supply voltages. According to the simulations reported in the previous section, looking closely at Table 4 and Figure 7, the following results were found for the five different materials and air in the space between the intermediate and primary electrodes.

### 4.1. Unipolar

Looking at the first column for the unipolar mode at W_S_ = 3 W_E_ (star graph in Figure 7a), the fixed V_P_ primary electrode is reduced one-fifth from 5 V_S_ to V_S_ from air to Rogers 3210. In the same way, V_P_ is reduced from 4.3 V_S_ to 0.9 V_S_ when W_S_ = 18 W_E_. Interestingly, as the dielectric constant grows, the difference between the V_P_ for W_S_ = 3 and 18 W_E_ reduces.

### 4.2. Bipolar

For the bipolar mode, V_P_ is reduced from 6 V_S_ to 1.6 V_S_ from ε = 1 to 10.2 for W_S_ = 3 W_E_. For W_S_ = 18 W_E_, V_P_ is reduced from 4.8 V_S_ to 1.4 V_S_. In a similar fashion to the unipolar mode, the two curves approach each other as the dielectric constant increases.

### 4.3. Maximum Displacement

Comparing W_S_ = 3 W_E_ and W_S_ = 18 W_E_ in this mode, the maximum displacement is higher for the latter, equal to 1.62 D_S_, which is double the former one with 1.30 D_S_. Similar to the unipolar and bipolar modes, the same trends occur as the primary electrode voltage is reduced from 4.4 Vs to 0.4 Vs for W_S_ = 3 W_E_ and from 5.0 V_S_ to 0.6 V_S_ for W_S_ = 18 W_E_ for materials from air to Rogers 3210. Using materials with a higher dielectric constant reduces the need for a high primary voltage (V_P_). However, this comes at the expense of needing a higher control voltage (resulting in a smaller FOM). Thus, the choice of material depends on the availability of the material and also on the design requirements, specifically with the ability to provide and control the varying V_I_ and fixed V_P_ voltages.

Looking at all the unipolar, bipolar, and maximum displacement modes discussed above, it can be seen that by employing solid gap-spacing materials in the D_2_ region with a dielectric constant of larger than 1, the required fixed primary electrode is lowered as the dielectric constant increases, as can be clearly seen in Figure 7. However, the FOM drops as the dielectric constant rises. This graph can be utilized to find the optimum fixed and varying voltage in our design cycle. Higher fixed voltages allow for lower control voltages. 

### 4.4. Numerical Results Verification

**Figure 8:**
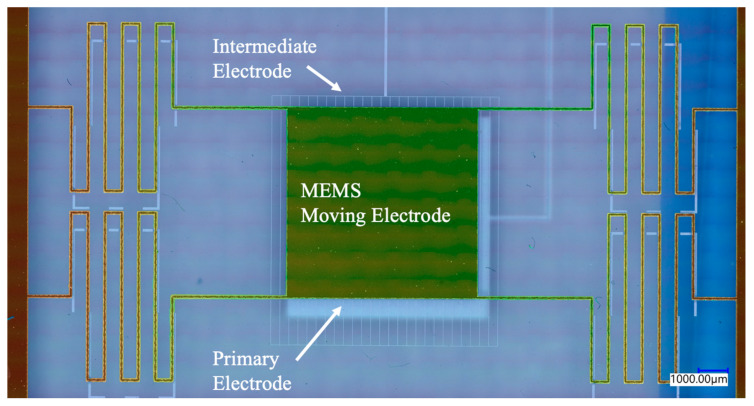
Photograph of the tri-electrode prototype actuator captured at 500× magnification using a Keyence VHX-7100 microscope.

**Figure 9:**
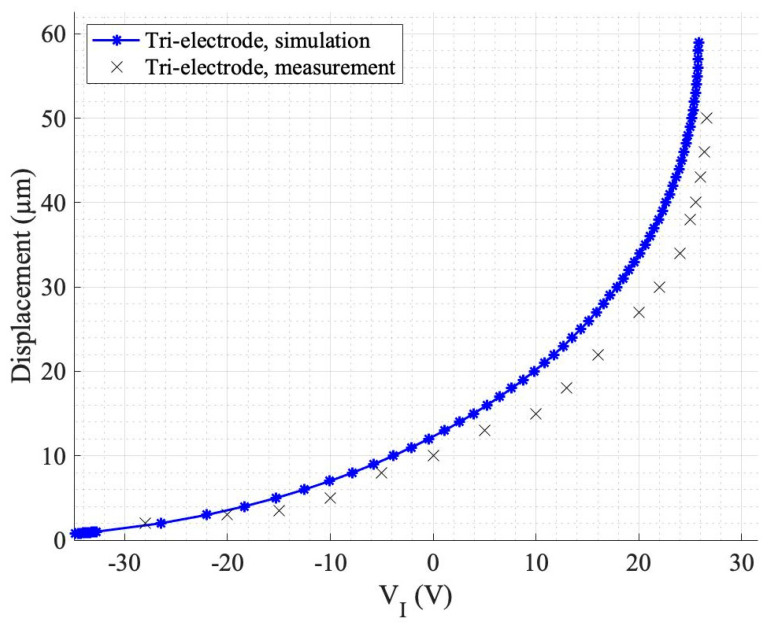
Displacement vs. control voltage (V_I_) for the fabricated actuator.

The numerical studies in this work build upon experimentally verified simulations of a device with quartz material in the gap between the intermediate and the primary electrodes [28]. However, the experimental simulator did not involve an optimized tri-electrode design to enable the best FOM performance, and rather was created to verify the numerical simulation method. A single-sided polished quartz wafer, with a dielectric constant of 3.82, was used as a spacing material (in the D_2_ region) to facilitate the fabrication of the prototype tri-electrode actuator. Figure 8 shows the prototype tri-electrode actuator with a square-shaped moving electrode and four serpentine springs fabricated in the Nanosystem Fabrication Lab at the University of Manitoba. The picture was captured using a Keyence (VHX-7100) (Osaka, Japan) microscope. Figure 9 illustrates the unipolar response curve, showing the displacement of the MEMS moving electrode versus the control voltage V_I_, and the close agreement between the numerical simulations and the experiment. The actuator design parameters are reported in Table 5.

## 5. Conclusions

This study investigated the performance impact of solid materials in the gap space between the driving electrodes of a tri-electrode actuator. Through charge analysis, the surface charge density on each of the three electrodes was extracted, revealing promising results. It was concluded that as the intermediate electrode voltage increases, more energy is transferred from the primary electrode to the MEMS, reducing the energy that can be dissipated between the fixed voltages. Additionally, numerical analysis of response curves and performance characteristics extractions indicated that using a material with a higher dielectric constant in the gap region between the intermediate and primary electrodes reduced the required primary electrode voltage. It was found that the higher the dielectric constant, the greater the reduction in the required primary electrode voltage. However, it is important to consider that a higher dielectric constant results in a smaller figure of merit (FOM), necessitating a larger control voltage. Therefore, in the design phase, it is important to balance the need for a low required primary voltage (V_P_) with the tolerance for a high varying intermediate control voltage (V_I_).

## Figures and Tables

**Table 1:** Tri-electrode topology design parameters.

Parameter	Description
D_1_	Gap between MEMS and intermediate electrode
D_2_	Gap between primary and intermediate electrode
W_S_	Perforation width of the intermediate electrode
W_E_	Electrode width of the intermediate electrode
V_P_	Voltage applied to the primary electrode (fixed voltage)
V_I_	Voltage applied to the intermediate electrode
ɛ	Dielectric constant of the material in D_2_ region
d	The amount of distance travelled by the MEMS electrode

**Table 2:** Tri-electrode configurations for the graph depicted in Figure 2.

Parameter	Value
D_1_	6 µm
D_2_	10 µm
W_S_	15 µm
W_E_	5 µm
V_P_	13.2 V
ɛ	2

**Table 3:** Materials with a dielectric constant ranging 1–10.2 used as the gap-spacing materials.

Material	Dielectric Constant	Ref.
Air	1	[31]
Microporous Polytetrafluoroethylene (PTFE)	1.5	[32]
Teflon	2	[33]
FR4	4.2	[34]
Glass	6.2	[35,36]
Rogers 3210	10.2	[37]

**Table 4:** FOM and maximum displacement normalized to conventional actuator characteristics (FOM_S_ and D_S_) with corresponding primary electrode voltage (V_P_). FOM_u_ and FOM_b_ are the FOM for unipolar and bipolar modes.

ε	FOM_u_/FOM_S_		FOM_b_/FOM_S_		Max. Displacement/D_S_	
	W_S_ = 3 W_E_	W_S_ = 18 W_E_	W_S_ = 3 W_E_	W_S_ = 18 W_E_	W_S_ = 3 W_E_	W_S_ = 18 W_E_
1	1.50 (V_P_ = 5.0 V_S_)	1.34 (V_P_ = 4.3 V_S_)	3.10 (V_P_ = 6.0 V_S_)	2.70 (V_P_ = 4.8 V_S_)	1.30 (V_P_ = 4.4 V_S_)	1.62 (V_P_ = 5.0 V_S_)
1.5	1.29 (V_P_ = 3.5 V_S_)	1.21 (V_P_ = 3.1 V_S_)	2.62 (V_P_ = 4.5 V_S_)	2.26 (V_P_ = 3.5 V_S_)	1.25 (V_P_ = 2.8 V_S_)	1.46 (V_P_ = 3.1 V_S_)
2	1.24 (V_P_ = 2.9 V_S_)	1.02 (V_P_ = 2.4 V_S_)	2.41 (V_P_ = 3.8 V_S_)	2.08 (V_P_ = 2.9 V_S_)	1.19 (V_P_ = 2.4 V_S_)	1.35 (V_P_ = 2.6 V_S_)
4.2	1.09 (V_P_ = 1.8 V_S_)	0.74 (V_P_ = 1.4 V_S_)	2.12 (V_P_ = 2.5 V_S_)	1.59 (V_P_ = 2.0 V_S_)	1.11 (V_P_ = 1.7 V_S_)	1.20 (V_P_ = 1.7 V_S_)
6.2	1.00 (V_P_ = 1.4 V_S_)	0.70 (V_P_ = 1.1 V_S_)	1.60 (V_P_ = 2.1 V_S_)	1.25 (V_P_ = 1.6 V_S_)	1.08 (V_P_ = 0.9 V_S_)	1.13 (V_P_ = 0.9 V_S_)
10.2 ± 0.2	0.85 (V_P_ = 1.0 V_S_)	0.64 (V_P_ = 0.9 V_S_)	1.20 (V_P_ = 1.6 V_S_)	1.12 (V_P_ = 1.4 V_S_)	1.04 (V_P_ = 0.4 V_S_)	1.08 (V_P_ = 0.6 V_S_)

**Table 5:** Tri-electrode design parameters for the graph depicted in Figure 9.

Parameter	Value
D_1_	140 µm
D_2_	490 µm
W_S_	300 µm
W_E_	16.7 µm
V_P_	109 V
ɛ	3.82

## Data Availability

Data are contained within the article.
